# Pap tests for cervical cancer screening test and contraception: analysis of data from the CONSTANCES cohort study

**DOI:** 10.1186/s12885-019-5477-8

**Published:** 2019-04-05

**Authors:** Stéphanie Mignot, Virginie Ringa, Solène Vigoureux, Marie Zins, Henri Panjo, Pierre-Jean Saulnier, Xavier Fritel

**Affiliations:** 1 Department of General Practice, University of Poitiers, France: 3 rue de la Milétrie, 86000 Poitiers, France; 2 CESP Centre for Research in Epidemiology and Population Health, U1018, Gender, Sexuality and Health team, University of Paris-Saclay, University of Paris-Sud, UVSQ, Villejuif, France; 3 Ined, Paris, France; 4 Obstetrics & Gynecology Department, Hôpital Bicêtre, GHU Sud, AP-HP, Faculty of Medicine, University of Paris Sud, F-94276 Le Kremlin Bicêtre, France; 5 Epidemiological Population-Based Cohorts Unit, INSERM UMS 11, Villejuif, France; 6 University of Paris-Descartes, Paris, France; 7 Clinical Research Centree CIC1402, INSERM, Department of Obstetrics, Gynecology, and Reproductive Medicine Poitiers University Hospital Centre, University of Poitiers, Poitiers, France

**Keywords:** Contraception, Pap test, Cervical cancer screening, Adhesion to screening

## Abstract

**Background:**

In France**,** a Pap test for cervical cancer screening is recommended every three years for all sexually active women aged 25 to 65 years. Modes of contraception (any or no contraception, with or without a visit to a physician, and with or without a gynecological examination) may influence adhesion to screening: women who use intrauterine device (IUD) should be more up to date with their cervical cancer screening more often than those using other means of contraception. Our objectives were to analyze the association between modes of contraception and Pap tests for screening.

**Methods:**

This cross sectional study is based on the CONSTANCES cohort enabled us to include 16,764 women aged 25–50 years. The factors associated with adhesion to cervical cancer screening (defined by a report of a Pap test within the previous 3 years) was modeled by logistic regression. Missing data were imputed by using multiple imputations. The multivariate analyses were adjusted for sex life, social and demographic characteristics, and health status.

**Results:**

Overall, 11.2% (1875) of the women reported that they were overdue for Pap test screening. In the multivariate analysis there was no significant difference between women using an IUD and those pills or implant of pap test overdue ORa:0.9 CI_95%_ [0.8–1.1], ORa 1.3 CI_95%_ [0.7–2.7] respectively. Women not using contraceptives and those using non-medical contraceptives (condoms, spermicides, etc.) were overdue more often ORa: 2.6 CI_95%_ [2.2–3.0] and ORa: 1.8 CI_95%_ [1.6–2.1] respectively than those using an IUD.

**Conclusion:**

Women seeing medical professionals for contraception are more likely to have Pap tests.

## Background

In many countries (including Canada, the United Kingdom, Belgium, Ireland, Italy, and France), public health authorities recommend cervical cancer screening (CCS) by a Pap test every 3 years for all sexually active women aged 25 to 65 years [1–3]. In France, 40 to 50% of the women in this population undergo screening irregularly or not at all (compared with the US, where only 17% of women reported that they were overdue for their Pap test) [1, 4]. CCS in France is mainly opportunistic, since organised screening, with invitations sent to women regularly exists in only 13 of 104 districts, among the 2.4 million women aged 25–65 years (13.4% of the total target population in France), 1,319,660 were invited for screening [5]. Gynaecologists (80%) and midwives perform most Pap tests; general practitioners do 11% [6]. Progressive extension of organised screening throughout France is scheduled to begin in 2018. The Pap test is covered by health insurance; in most cases; women must pay the doctor and the laboratory in advance for the test, but will be reimbursed, usually within two weeks. Pap tests are free of charge, with no money advanced by the woman, in organised screening programmes and for women with very low incomes.

Organised screening programmes reduce the incidence of cervical cancer [7]. It is important to explore the factors that influence adherence to screening, for even with organised programmes, some women may be missed or may not participate. Factors that may reduce cancer screening rates include low educational level, country of birth, poor self-reported health status, and comorbidities (e.g., diabetes or obesity) [8, 9]. Other risk factors for overdue (or non-existent) screening status include being a foreigner or of immigrant origin [10]. Although seeing a general practitioner (GP) is positively associated with Pap testing [11], patients of obstetrician-gynaecologists have higher CCS rates than those of other physicians [12]. Besides these characteristics, women’s sex lives, including their sexual orientation, also influence Pap test adherence. Lesbians are less prevention-oriented in their health care behaviour than heterosexual women and appear to avoid or at least miss routine screening tests such as Pap smears [13].

Of the factors potentially associated with women’s adherence to this screening, mode of contraception has mainly been studied for single types of contraception in any given study [1, 14]. Some contraceptives require a doctor’s prescription; these include contraceptive pills, intrauterine devices (IUDs), and implants. Other methods (condom, natural spermicides, the rhythm, or Knaus-Ogino method)^1^ do not. Means of contraception that require a medical visit for their use (prescription or placement) share with Pap tests the need for familiarity with the healthcare system and comfort in understanding how it functions, that is, the ability to make the necessary appointments and have the procedures performed. More generally, both require adherence to preventive practices.

We hypothesised that the woman’s opportunity to have a Pap test may be influenced by the mode of contraception she uses and, more precisely, that her visit to her doctor for the prescription or follow-up of contraception promotes CSS. The visit for contraception may be the opportunity for women to be offered this cancer screening. For example, GPs and other primary care physicians probably find it easier to raise the subject of Pap tests at a consultation for contraception than during visits for other health problems unrelated to sexual health or reproduction.

The placement of an intrauterine device (IUD) requires a gynaecological examination, with a speculum. In Europe as in the United States, it is gynaecologists who most often place IUDs [1, 15]. Use of a speculum for this placement facilitates performance of a Pap test, unlike visits for other means of contraception, which do not require gynaecological examinations. This point may explain why patients of obstetrician-gynaecologists have higher cervical cancer screening rates than patients of other physicians [16]. Accordingly, women who use an IUD should more often be up to date with their Pap test than women who use other means of contraception (implant, pills, condom, natural spermicides, and the rhythm method). Our objective here is to analyse the association between contraceptive practices, according to whether they require visits to a healthcare professional or not, and screening status (overdue, defined as a last Pap smear more than 3 years ago, or up-to-date, essentially a Pap smear that is not overdue) and simultaneously take into account other characteristics that might affect screening use.

## Methods

### Study population

This cross sectional study is based on the CONSTANCES cohort which includes volunteers aged 18–69 years who undergo health tests at inclusion in 22 selected health screening centres from the principal regions of France. These participants are randomly selected from adults covered by the National Health Insurance Fund (i.e., salaried workers, currently working or retired and their family), stratified for age, sex, region and socioeconomic position. CONSTANCES collects data on personal, environmental, behavioural, occupational and social factors from self-administered questionnaires at inclusion and annually thereafter, mailed to and returned by participants (or collected in the health centres). This is a general-purpose epidemiological cohort designed to study a wide range of health problems in various disciplines in the general population. Its long-term objective is to follow 200,000 members (men and women) of the French population, aged 18 to 69 years; inclusion in this cohort began in 2012 [17]. After enrolment, participants are followed up by an annual self-administered questionnaire sent to their homes (paper or web-based), and they are invited for a new health examination every 5 years. The data considered here were collected at inclusion and come from the questionnaires about lifestyle, women’s health, and occupational exposures. The data were collected from 2012 to 2015. Body mass index (BMI) was obtained from participants’ weight and height measurements, collected at the initial medical examination.

This analysis covers the women aged 25 to 50 years recruited between January 2012 and 2015. It excluded the following women: those older than 50 years; women younger than 50 years who reported that they were in menopause, either spontaneously or after surgery (hysterectomy or bilateral oophorectomy); those who reported they have never had sex; those who reported that they have had cervical cancer; those who required an annual Pap test for health reasons related to an immune system deficiency; pregnant women and those not using contraceptives because they were trying to become pregnant when they completed the questionnaire.

### Outcome measurements

The outcome measure, being overdue for a Pap test, was based on self-report. The items in the women’s health questionnaire referring to the Pap test were: “Have you ever had one or more Pap tests (smears taken from the cervix): yes/no. If yes, when was the last one?” The overdue status was defined by no Pap test in the past 3 years (day/month/years). If the women reported a Pap test during the past 3 years they were considered “up to date”.

#### Exposure measurement

For contraception, the principal explanatory variable, we distinguished the different types of contraceptives according to their degree of medicalisation *(does or does not require prescription and follow-up by a healthcare professional)* and then in more detail, into 5 contraceptive choices:

- Contraception by IUD. This device must be placed by a physician (or midwife) and changed every 3 to 10 years. Women who use it are advised to have an annual clinical examination. We hypothesised that the women using it would be at the lowest risk of overdue status.
- Combined oestrogen-progestin or progestin-only contraceptives, regardless of their form (oral, patch, ring, injection), which require at least one medical consultation annually for their prescription.
- Contraception by implants. These must be placed by a physician (or midwife) and changed every 3 years.
- Non-medicalised contraception. These are the contraceptives that require no medical visit (condom, spermicides, natural or rhythm methods). We include permanent contraception in this category because once it has been performed the woman no longer needs to return to see the physician for contraception. The type of permanent contraception (male or female) was not specified in the questionnaire, but previous data suggest it was most probably permanent female contraception [18].
- Absence of contraception. This category included the women who reported sexual relations but not contraceptive use, although they did not want to become pregnant.

### Sex life

Women answered the following questions about their sex lives and reproduction: sex of partners (male/female/both/do not wish to answer (DNWA)), number of lifetime partners (number/DNWA), new partner in the past 12 months (yes/no/DNWA), pain during intercourse (never/sometimes/often/always), sexual satisfaction (currently your sex life seems: not at all satisfactory/not very satisfactory/satisfactory/very satisfactory /DNWA/not applicable). The response to this question was summarised as satisfactory and unsatisfactory, with women who answered “very satisfactory or satisfactory” classified as satisfied. The women considered to have pain during intercourse (dyspareunia) were those who answered “often” or “always”.

### Social and demographic characteristics

Age was categorised in 3 classes (> 25–29 years, 30–44 years, and 45–50 years), a division based essentially on the lifetime periods of contraception observed among women in France. Before the age of 30 (and their first pregnancy), women in France tend to use birth control pills. From 30 years to 45, they tend to use an intrauterine device (IUD), and starting around the age of 45, the percentage of women not using contraception increases [19].

The indicators of social position considered were: socio-occupational category, defined by current occupation or the occupation practiced longest for women not working at the time of the survey. Socio-occupational category was coded according to the 2003 INSEE (national institute of statistics and economic studies) classification [20].

Educational level was defined by the highest diploma completed: less than the baccalaureate or school-leaving exam (“bac”), post-secondary degree, more advanced degrees.

Socioeconomic situation was defined by 2 variables: has ever foregone medical care for herself or one of her children and has or has had financial difficulties (in the past, currently but only recently, currently and for a long time, no financial difficulty ever).

The other indicators considered were self-reported parity (nulliparous: no child; primiparous: at least one child; multiparous: more than one child), civil status (age, family situation), and geographic origin, defined according to place of birth.

### Health status

A specific question allowed respondents to classify their health status as good, medium, or poor.

The categories for smoking were: current smoker, ex-smoker, non-smoker; for alcohol consumption: irregular consumption (less than 4 times a month), regular consumption (one to several times a week), and not currently; for marijuana use (yes/no/DNWA). The weight and height of each participant were measured at the medical examination at the health centre and enabled calculation of her body mass index (BMI). This variable was introduced in categories according to the WHO classification (< 18.5 underweight, 18.5–24.9 normal weight, 25.0–29.9 overweight, 30.0–39.9 obese, > 40 morbidly obese).

### Statistical analyses

Continuous variables (age, BMI) were described by their means and standard deviations, and the categorical variables as percentages. The continuous variables were then discretised into categories. To assess the association between overdue status and the categorical variables, we performed Chi2 tests.

To understand the role of each variable, we first studied the associations between the explanatory variables and overdue status in a univariate analysis. Variables were retained when they were associated with overdue status with a *P* value < 0.05. They were then included in 3 separate thematic logistic regressions (contraception and sex life, social and demographic characteristics, and health status). These models were simplified by backward elimination. A final model including the associated variables for each thematic model (at *P*< 0.05) also underwent the backward elimination procedure. The associations between overdue status and the different variables of interest were expressed by adjusted odds ratios and their 95% confidence intervals.

Missing data were imputed by using multiple imputations with fully conditional specification (SAS 2013) and assuming missingness at random (MAR). To make the MAR assumption more plausible, every previously described variable was used for the imputation model [21, 22], including the outcome. Excluding the outcome from the imputation model could have hidden some associations, and including it did not change the standard deviations [23]. Ten complete datasets were created. This method, known as MID (multiple imputations, then deletion), uses information about the dependent variable in the imputation model as does the standard imputation method, but cases with imputed outcomes are deleted before analyses [24]. Overall, there was less than 10% missing data for all variables except educational level (10.8%), socio-occupational categories (12%), foregone medical care (27%), sexual orientation (12.5%), pain during sexual intercourse (14%), satisfactoriness of sex life (18.9%), number of lifetime partners (49.8%), new partner in the past 12 months (13%), perceived health status (11.0%), smoking status (11.5%), marijuana use (11.7%) and alcohol consumption (13.9%). The analyses were performed with SAS software, version 9.4.

### Ethics

The national council on statistical information (CNIL) approved the CONSTANCES study before it began (CNIL authorisation n°910,486), as well as an additional related application CNIL authorisation n°1,881,675).CONSTANCES was approved by the National Council for Statistical Information (Conseil national de l’information statistique-CNIS), the National Medical Council (Conseil national de l’Ordre des médecins-CNOM), the Institutional Review Board of the National Institute for Medical Research (INSERM) and our local Committee for Persons Protection (Comité de protection des personnes).

## Results

**Fig. 1:**
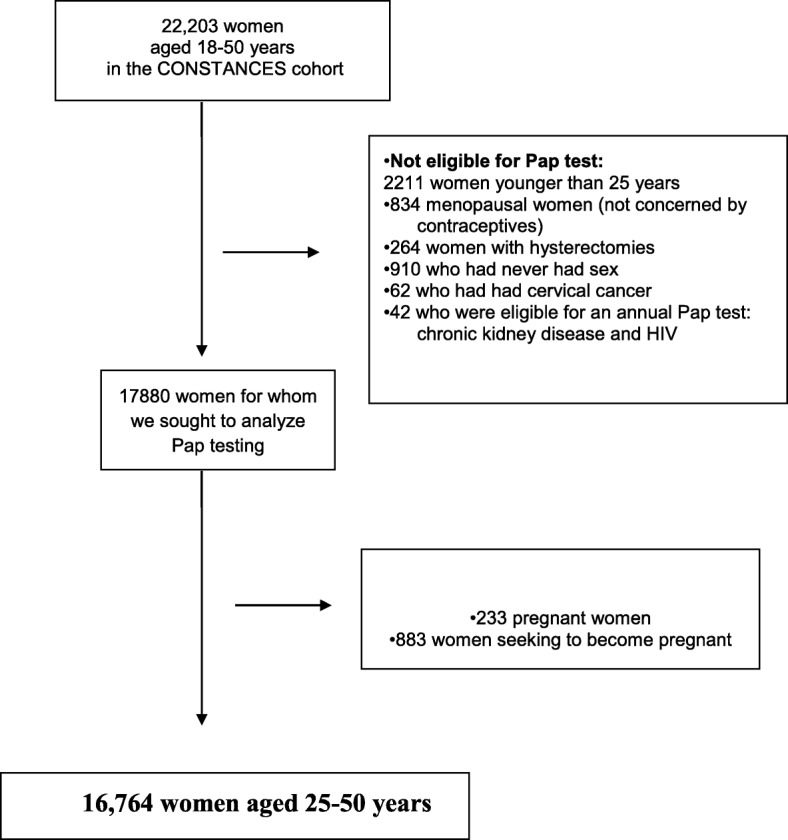
Selection of the sample to be analyzed

In all, 22,203 women responded to the CONSTANCES questionnaires used for our analysis. Women were excluded due to age (< 25 years or > 50), menopausal status, had never had sex, or a medical history of hysterectomy, cervical cancer, chronic kidney disease, or HIV (Fig. 1). The date of the Pap test could not be determined for 1736 of them (information missing or clearly erroneous). The women who did not answer the Pap test questions were younger (15.9% vs 12.5% younger than 30 years), less well educated (17.3% vs 12.6% left school without a baccalaureate, *P*< 0.001), and had more often foregone medical care (26.0% vs 22.4%). The responders did not differ in their response rates to the questions about contraception, sexual orientation or other questions about their sex lives from the no responders (Table 1).

**Table 1:** Differences between responders and non-responders to the pap question

	Responders	No responders	Total	
	% (*n*)	% (*n*)	% (*N*)	*P*
Age groups				0.002
25–29	15.9 (2336)	12.5 (190)	15.6 (2526)	
30–44	58.6 (8595)	60.8 (924)	58.8 (9519)	
45–50	25.5 (3746)	26.7 (405)	25.6 (4151)	
Total	100.0 (14677)	100.0 (1519)	100.0 (16196)	
Educational level				< 0.001
Below Baccalaureate	12.6 (1644)	17.3 (234)	13.0 (1878)	
Baccalaureate	15.0 (1969)	17.8 (240)	15.3 (2209)	
Post-secondary school	71.2 (9313)	63.9 (862)	70.5 (10175)	
Other	1.2 (163)	1.0 (13)	1.2 (176)	
Total	100.0 (13089)	100.0 (1349)	100.0 (14438)	
Financial difficulties				0.017
Never	59.1 (7850)	55.2 (761)	58.8 (8611)	
In the past	24.4 (3235)	25.5 (351)	24.5 (3586)	
Currently	9.3 (1233)	10.7 (147)	9.4 (1380)	
For a long time	7.2 (957)	8.7 (120)	7.3 (1077)	
Total	100.0 (13275)	100.0 (1379)	100.0 (14654)	
Contraception				0.349
Hormonale	29.8 (4373)	27.7 (421)	29.6 (4794)	
UID	31.3 (4594)	31.9 (485)	31.4 (5079)	
Non-medical(condom, natural, etc)	22.7 (3331)	22.5 (342)	22.7 (3673)	
No contraception	15.8 (2326)	17.4 (265)	16.0 (2591)	
Implant	0.4 (53)	0.4 (6)	0.4 (59)	
Total	100.0 (14677)	100.0 (1519)	100.0 (16196)	
Socio-professional categories				< 0.001
Farmer	0.1 (7)	0	0.0 (7)	
Tradesperson, shopkeeper	1.1 (142)	1.6 (21)	1.1 (163)	
Manager, professional	27.0 (3484)	20.9 (282)	26.4 (3766)	
Intermediate professional	29.1 (3755)	28.1 (380)	29.0 (4135)	
Office, sales, and service staff	34.4 (4441)	37.6 (508)	34.7 (4949)	
Blue-collar	2.7 (342)	4.5 (61)	2.8 (403)	
Never worked	0.9 (119)	1.3 (18)	1.0 (137)	
Other	4.7 (608)	5.9 (80)	4.8 (688)	
Total	100.0 (12898)	100.0 (1350)	100.0 (14248)	
Body mass index				0.313
< 18	12.4 (1816)	11.5 (175)	12.3 (1991)	
18–25	61.1 (8959)	59.6 (904)	60.9 (9863)	
25–30	17.8 (2611)	19.5 (296)	18.0 (2907)	
30–40	8.0 (1167)	8.4 (128)	8.0 (1295)	
> 40	0.8 (113)	1.0 (15)	0.8 (128)	
Total	100.0 (14666)	100.0 (1518)	100.0 (16184)	
New partner in the past 12 months				0.418
Yes	13.0 (1658)	13.7 (181)	13.0 (1839)	
No	87.0 (11141)	86.3 (1136)	87.0 (12277)	
Total	100.0 (12799)	100.0 (1317)	100.0 (14116)	
Marijuana use				0.257
Yes	41.8 (5417)	40.2 (546)	41.6 (5963)	
No	58.2 (7542)	59.8 (812)	58.4 (8354)	
Total	100.0 (12959)	100.0 (1358)	100.0 (14317)	
Alcohol consumption				0.716
Drinks alcohol regularly	50.0 (6325)	48.9 (633)	49.9 (6958)	
Drinks alcohol irregularly	44.5 (5620)	45.3 (587)	44.5 (6207)	
Do not drink alcohol	5.5 (698)	5.8 (75)	5.5 (773)	
Total	100.0 (12643)	100.0 (1295)	100.0 (13938)	
Smoking status				0.282
Smoker	23.2 (3010)	24.5 (332)	23.3 (3342)	
Non-smoker	76.8 (9982)	75.5 (1025)	76.7 (11007)	
Total	100.0 (12992)	100.0 (1357)	100.0 (14349)	
Perceived health status				< 0.001
Good	81.3 (10619)	76.4 (1026)	80.8 (11645)	
Medium	15.1 (1977)	19.4 (260)	15.5 (2237)	
Poor	3.6 (467)	4.2 (57)	3.6 (524)	
Total	100.0 (13063)	100.0 (1343)	100.0 (14406)	
Parity				0.012
Nulliparous	28.7 (4068)	25.2 (367)	28.3 (4435)	
Primiparous	17.9 (2547)	19.9 (290)	18.1 (2837)	
Multiparous	53.4 (7583)	54.8 (797)	53.5 (8380)	
Total	100.0 (14198)	100.0 (1454)	100.0 (15652)	
Civil status				0.758
Single	34.3 (4570)	35.0 (484)	34.4 (5054)	
Maried or civil union	55.8 (7429)	54.7 (757)	55.7 (8186)	
Sépared, divorced, widowed	9.9 (1323)	10.3 (142)	10.0 (1465)	
Total	100.0 (13322)	100.0 (1383)	100.0 (14705)	
Sex life satisfactory				0.201
No	32.4 (3872)	34.3 (410)	32.6 (4282)	
Yes	67.6 (8065)	65.7 (787)	67.4 (8852)	
Total	100.0 (11937)	100.0 (1197)	100.0 (13134)	
Pain during sexual intercourse/dyspareunia				0.609
Never	93.3 (11800)	92.9 (1205)	93.2 (13005)	
Often	6.7 (850)	7.1 (92)	6.8 (942)	
Total	100.0 (12650)	100.0 (1297)	100.0 (13947)	
Sexual orientation				0.926
Heterosexual	98.2 (12611)	98.3 (1310)	98.2 (13921)	
Lesbian	1.8 (226)	1.7 (23)	1.8 (249)	
Total	100.0 (12837)	100.0 (1333)	100.0 (14170)	
Number of lifetime partners				0.855
Fewer than 6	59.7 (4426)	59.3 (420)	59.7 (4846)	
6 to 29	39.2 (2904)	39.4 (279)	39.2 (3183)	
30 to 50	1.1 (78)	1.3 (9)	1.1 (87)	
Total	100.0 (7408)	100.0 (708)	100.0 (8116)	
Has foregone medical care				
Yes	22.4 (2392)	26.0 (306)	22.7 (2698)	
No	77.6 (8302)	74.0 (872)	77.3 (9174)	
Total	100.0 (10694)	100.0 (1178)	100.0 (11872)	
Geographic origin				0.52
Metropolitain France	89.5 (12008)	89.0 (1252)	89.5 (13260)	
French overseas departments and territories	0.8 (111)	1.3 (18)	0.9 (129)	
Europe	4.0 (539)	3.8 (54)	4.0 (593)	
Asia/Africa	4.2 (567)	4.4 (62)	4.2 (629)	
Other	1.4 (189)	1.4 (20)	1.4 (209)	
Total	100.0 (13414)	100.0 (1406)	100.0 (14820)	

We conducted an initial analysis without imputation for the missing data. A sensitivity analysis was then performed with 10 imputations for all missing variables, including outcome. The results for associations between the variables were similar (accordingly, we present only the results with imputations).

The overdue rates before and after imputation were of the same order (respectively 12.5% vs 13.5 to 15.6%) (Table 2).

**Table 2:** Comparaison between the rate of overdue with and without imputation

		Up to date		Overdue		Total
		*N*	%	*N*	%	*N*
Number of imputation	Imputed					
1	No	13,153	87.52	1875	12.48	15,028
	Yes	1465	84.39	271	15.61	1736
	Total	14,618	87.20	2146	12.80	16,764
2	Imputed					
	No	13,153	87.52	1875	12.48	15,028
	Yes	1469	84.62	267	15.38	1736
	Total	14,622	87.22	2142	12.78	16,764
3	Imputed					
	No	13,153	87.52	1875	12.48	15,028
	Yes	1489	85.77	247	14.23	1736
	Total	14,642	87.34	2122	12.66	16,764
4	Imputed					
	No	13,153	87.52	1875	12.48	15,028
	Yes	1493	86.00	243	14.00	1736
	Total	14,646	87.37	2118	12.63	16,764
5	Imputed					
	No	13,153	87.52	1875	12.48	15,028
	Yes	1470	84.68	266	15.32	1736
	Total	14,623	87.23	2141	12.77	16,764
6	Imputed					
	No	13,153	87.52	1875	12.48	15,028
	Yes	1465	84.39	271	15.61	1736
	Total	14,618	87.20	2146	12.80	16,764
7	Imputed					
	No	13,153	87.52	1875	12.48	15,028
	Yes	1500	86.41	236	13.59	1736
	Total	14,653	87.41	2111	12.59	16,764
8	Imputed					
	No	13,153	87.52	1875	12.48	15,028
	Yes	1468	84.56	268	15.44	1736
	Total	14,621	87.22	2143	12.78	16,764
9	Imputed					
	No	13,153	87.52	1875	12.48	15,028
	Yes	1484	85.48	252	14.52	1736
	Total	14,637	87.31	2127	12.69	16,764
10	Imputed					
	No	13,153	87.52	1875	12.48	15,028
	Yes	1502	86.52	234	13.48	1736
	Total	14,655	87.42	2109	12.58	16,764

This analysis finally included 16,764 women aged 25–50 years. Their mean age was 39.0 ± 7.3 years. Overall, 11.2% (1875) of the women reported that they were overdue for Pap test screening: 4.1% (683) had never had this test and 7.1% (1192) had last one more than 3 years ago.

### Factors associated with overdue status, univariate analysis and multivariate analysis

In the univariate analysis, we observed an association between some contraceptive practices and overdue status for a Pap test: the highest risk of women overdue (OR 3.2, 95% CI 2.8–3.7) were in the group not using contraceptives. In turn, women with an implant were overdue more often (OR 2.6, 95% CI 1.4–4.7) than those using hormonal contraception (OR 1.2, 95% CI 1.0–1.4), and those with an IUD (reference) were least likely to be overdue (Table 3).

**Table 3:** Overdue Pap test status (women aged 25 to 50 years); univariate analysis and multivariate analysis Results pooled from 10 imputed datasets (*N* = 16,764)

	OR [95% CI]	*P*	aOR [95% CI]	*P*
Contraceptive Practices				
Hormonal contraception	1.2 [1.0–1.4]	0.05	0.9 [0.8–1.1]	0.4
Implant	2.6 [1.4–4.7]	0.0024	1.3 [0.7–2.7]	0.4
IUD	Réf,	–	Réf,	–
Non-medical (condom, natural,etc)	2.1 [1.8–2.4]	< 0.0001	1.8 [1.6–2.1]	< 0.0001
No contraception	3.2 [2.8–3.7]	< 0.0001	2.6 [2.2–3.0]	< 0.0001
Socioeconomic and demographic characteristics				
Age groups				
25–29 years	1.6 [1.4–1.8]	< 0.0001	1.6 [1.3–2.0]-	< 0.0001
30–44 years	1.1 [1.0–1.3]	0.04	1.3 [1.1–1.5]	0.0003
45–50 years	Réf,	–	–	–
Geographic origin				
metropolitan France	Réf,	–	Réf,	–
French overseas departments and territories	2.9 [1.8–4.7]	< 0.0001	2.1 [1.3–3.5]	< 0.004
Europe	3.7	15.6	1.3 [1.0–1.7]	0.02
Africa/Asia	2.7 [2.2–3.2]	< 0.0001	1.8 [1.5–2.2]	< 0.0001
Other	1.3	15.9	1.1 [0.7–1.7]	0.60
Educational level				
Below Baccalaureate	1.8 [1.5–2.0]	< 0.0001	1.3 [1.0–1.5]	0.18
Baccalaureate	1.3 [1.1–1.5]	0.0005	1.1 [0.9–1.3]	0.25
Post-secondary school	Réf,	–	Réf,	–
Other diplomas	1.0 [0.7–1.5]	0.90	0.8 [0.5–1.3]	0.40
Civil status				
Single	1.6 [1.5–1.8]	< 0.0001	1.2 [1.1–1.4]	0.02
Married or civil union	Réf,	–	Réf,	–
Separated, divorced, widowed	1.3 [1.1–1.5]	0.006	1.1 [0.9–1.3]	0.37
Parity				
Nulliparous	1.7 [1.5–1.9]	< 0.0001	1.1 [0.9–1.4]	0.18
Primiparous	1.1 [1.0–1.3]	0.06	0.9 [0.8–1.0]	0.11
Multiparous	Réf,	–	Réf,	–
Socio-profesional categories				
Farmer	0	0	–	
Tradesperson, shopkeeper	1.2 [0.8–2.0]	0.40	1.1 [0.6–1.8]	0.84
Manager, professional	Réf,	–	Réf,	–
Intermediate professional	1.0 [0.9–1.2]	0.95	1.0 [0.9–1.2]	0.7
Office, sales, and service staff	1.4 [1.2–1.6]	< 0.0001	1.3 [1.2–1.5]	< 0.0001
Blue-collar	2.6 [2.1–3.3]	< 0.0001	1.7 [1.3–2.3]	0.0005
Never worked	3.8 [2.8–5.2]	< 0.0001	1.9 [1.4–2.6]	0.0002
Other	1.5 [1.1–1.9]	0.73	1.1 [0.8–1.4]	0.70
Has foregone medical care				
Yes	1.9 [1.6–2.1]	< 0.0001	1.3 [1.0–1.5]	0.02
No	Réf,	–	Réf,	–
Financial difficulties				
In the past	1.4 [1.2–1.6]	< 0.0001	1.1 [1.0–1.3]	0.12
Currently	1.9 [1.6–2.3]	< 0.0001	1.1 [0.8–1.2]	0.83
For a long time	1.6 [1.3–1.9]	< 0.0001	1.1 [0.8–1.4]	0.52
Never	Réf,	–	Réf,	–
Sexual function				
Sexual orientation				
Lesbian	2.3 [1.7–3.1]	< 0.0001	1.8 [1.4–2.4]	< 0.0001
Heterosexual	Réf,	–	Réf,	–
Pain during sexual intercourse/dyspareunia				
Often	1.3 [1.1–1.7]	0.011	1.0 [0.8–1.3]	0.66
Never	Réf,	–	Réf,	–
Sex life satisfactory				
Yes	Réf,	–	Réf,	–
No	1.2 [1.1–1.4]	< 0.0001	1.2 [1.1–1.4]	0.0002
Number of lifetime partners				
Fewer than 6	Réf,	–	–	–
6 to 29	1.3 [0.9–2.1]	0.19	–	**–**
30 to 50	0.9 [0.8–1.0]	0.11	–	**–**
New partner in the past 12 months				
Yes	1.2 [1.1 1.4]	0.0048	0.9 [0.7–1.0]	0.13
No	Réf,	–	Réf,	–
Health status				
Perceived health status				
good	Réf,	–	Réf,	–
medium	1.6 [1.4–1.8]	< 0.0001	1.1 [1.0–1.3]	0.11
poor	2.0 [1.6–2.6]	< 0.0001	1.2 [1.0–1.6]	0.09
Body mass index				
BMI < 18 malnourished/underweight	1.0 [1.0–1.3]	0.17	1.0 [0.8–1.1]	0.56
18 < =BMI < 25 normal	Réf,	–	Réf,	–
25 < =BMI < 30 overweight	1.3 [1.1–1.4]	0.0004	1.2 [1.0–1.4]	0.014
30 < = BMI < 40 obese	2.0 [1.7–2.3]	< 0.0001	1.6 [1.4–1.9]	< 0.0001
BMI > 40 morbidly obese	2.5 [1.6–3.9]	0.0001	1.6 [1.0–2.6]	0.067
Smoking status				
Smoker	1.3 [1.1–1.4]	0.0002	1.1 [1.0–1.3]	0.06
Non-smoker	Réf,	–	Réf,	–
Marijuana use				
Yes	0.9 [0.9–1.0]	0.25	–	–
No	Réf,	–	–	–
Alcohol consumption				
Drinks alcohol regularly	0.9 [0.8–1.0]	0.026	1.0 [0.9–1.1]	0.77
Drinks alcohol irregularly	Réf,	–	Réf,	–
Do not drink alcohol	1.4 [1.2–1.8]	0.001	1.2 [0.9–1.5]	0.13

*Variables where the only response possible was affirmative. The women who did not check the box were considered not to have the condition mentioned

Adjsutement had been done on all variable of the model

Compared with the women whose status was up to date, the women who were overdue were also younger, more often born abroad and less well educated. Women who were blue-collar workers and those who reported they did not work, as well as those reporting financial difficulties, were also overdue more often. This was also true of women who were overweight or obese (Table 3).

Among the women who had had at least one Pap test, those seeing only a GP were overdue more often than those seeing a gynaecologist (14% vs 7.3%, *P*< 0.0001). Gynaecologists performed the Pap test most often (82.5%); only 1.8% of the samples were taken by a clinical pathologist or a midwife.

In the multivariate analysis women not using contraceptives (aOR 2.6, 95% CI 2.2–3.0) and those using non-medical contraceptives (aOR 1.8, 95% CI 1.6–2.1) were overdue more often than those with an IUD (Table 3). There was no difference in the overdue rate among the women with different types of medicalised contraception (hormonal contraception aOR 0.9, 95% CI 0.8–1.1; Implant aOR 1.3 95% CI 0.7–2.7). The social and demographic characteristics associated with an overdue status were the same as those found in the univariate analysis. Smokers were at the highest risk of this status. Long-term financial difficulties (aOR 1.3, 95% CI 1.0–1.6), overweight (aOR 1.2, 95% CI 1.0–1.4) and obesity (aOR 1.6,95% CI 1.0–2.6), and homosexuality (aOR 1.8, 95% CI 1.4–2.4) were all associated with overdue status.

## Discussion

Our analysis of data from the CONSTANCES cohort shows that, contrary to our hypothesis, Pap test uptake did not differ between women using medicalised hormonal contraception (pills or implant) and those using an IUD. On the other hand, the women who used no contraception and those who used non-medical contraception were, as we had supposed, overdue for Pap tests more often than all the other women.

### Strengths and weaknesses

The CONSTANCES study population has several strengths: population diversity, a very large sample size, a great variety of questions that enabled us to explore the association between Pap test CCS and contraceptive choices, while taking into account health factors such as BMI, economic factors such as financial problems, and those associated with sexual life. In France, questions concerning religion cannot be asked without specific authorisation; hence the survey did not collect information about women’s religious practices. Religious beliefs can, however, play a role both in adhesion to CCS [25], because gynaecological examinations may be performed by a male physician or to respond to questions about sexual practices and behaviour. Women with higher educational levels were overrepresented in the sample: 24% of the women in our sample had completed a post-secondary degree, although such women account, according to INSEE, for only 14% of French women older than 15 years [20]. In addition, participation in the cohort requires at least a minimum reading level in French, which excludes foreigners who do not read French comfortably. We can assume that these women belong to a more disadvantaged population, potentially more distant from the health-care system.

The women from privileged environments and those who had university educations had the best screening coverage rates [26]. This may explain why the rate of women up-to-date for their Pap tests in our sample is higher than that found in health insurance data. Moreover, the sample we analysed was not weighted; it is difficult to assess the effects of selection because there are no representative data on this topic for France. The self-reported outcomes can result in misclassification bias and might have affected the associations. Women might have underestimated the date of their last Pap test (social desirability bias).

This study is a cross sectional study. Although it can suggest arguments for a causal relation between Pap tests and contraception, it cannot prove a causal link between outcome and exposure.

### Comparison with other studies

The women who used a contraceptive method requiring a medical prescription were less often overdue for Pap tests than those who used other means of contraception. These results are consistent with those of a study conducted in France (Rhône-Alpes region) [14] and of another in Norway in 2011 [27]. The women who were not using contraceptives at the moment of the survey, were not pregnant and did not wish to be probably saw health-care professionals less often than the others and therefore had fewer opportunities for a professional to offer them a Pap test. In the FECOND study conducted in the general population, Bajos et al. found that some of the women not using contraception reported that they “did not know where to go” to obtain a prescription for it [19]. These women might also be less concerned about their health and might globally receive less care from medical professionals. Financial difficulties might also play a role in the non-use of contraceptives and in the non-performance of the Pap test, both of which require that the patient pay in advance (before reimbursement) [28].

Contrary to our hypothesis, the overdue status of women who used an IUD was no better than that of the women using other medicalised forms of contraception. We can thus suppose that more than the need for a gynaecological examination, it is the meeting with the healthcare professional for the prescription that promotes Pap testing.

We observed that sexual orientation was associated with performance of Pap tests; these associations confirm findings in the literature [29] that lesbians have Pap tests less often and feel less concerned by this screening. Their lack of need for contraception may also diminish the likelihood of medical visits during which CCS might be proposed.

The Pap test, as currently performed, requires the placement of a speculum, which can be painful. Even when not painful, the fact that it requires a gynecologic examination may be a barrier to Pap tests both for women and for general practitioners. It can be more difficult than with the gynaecologist to undress or to raise questions related to sex; the GP is often the family physician and it might be thus more difficult to broach personal or intimate questions [30–32]. Women may also not know that GPs are able to perform Pap tests [33]. Some GPs making self-sampling kits available for women might facilitate this screening. Some GPs probably offer Pap smears when they are prescribing contraceptives; the women may be more likely to accept a gynaecological examination at that moment. Asking about contraception also might be an approach to opening a discussion.

about a Pap test and to mention that the GP can perform this test, and then to offering it. A still more promising.

method of facilitating this screening might be making self-sampling kits available for women. These kits enable.

women to take their own vaginal samples. Many studies have found that Human papillomavirus (HPV) self-sampling facilitates screening uptake among overdue women. A meta-analysis found higher participation in the self-sampling arm compared to the control arm when self-sampling kits were sent directly to women at their home address [34]. In a study, Lim Aw found that offering self-sampling to CCS non-attenders opportunistically in primary care is feasible [35]. A recent meta-analysis Arbyn M,found that offering self ampling kits generally is more effective in reaching underscreened women than sending invitations [36].

This method may be more acceptable for some women and for their GPs (less time spent and less discomfort). These kits are designed to detect HPV at high risk of carcinogenicity and have a sensitivity greater than 90% and a specificity greater than 98% [37].

## Conclusion

Our results show that women seeing medical professionals for contraception are more likely to have Pap tests. In countries where this screening is opportunistic, proposing it to all women who seek medical care for contraception or any other motive is necessary if we wish to improve the coverage of screening for cervical cancer. The screening can be performed by GPs, who see women for reasons other than contraception. Screening can be improved by increasing the percentage performed by these primary care physicians who see women most often.

## Abbreviations

BMI: Body mass index
CCS: Cervical cancer screening
CNIL: National Council on Statistical Information
DNWA: Do not wish to answer
GP: General pratitionner
HIV: Human immunodeficiency virus
HPV: Human papillomavirus
INSEE: French National Institute for Statistics and Economic Research
INSERM: Institutional Review Board of the National Institute for Medicale Research
MAR: Missingness at random
MID: Multiple imputations and deletion

## Footnotes

1 This method is a so-called “natural” calendar-based contraceptive method, better known as the rhythm method, which involves avoiding sexual intercourse during the period when the woman can become pregnant.
